# Clinical Characteristics and Management of Patients With McCune-Albright Syndrome With GH Excess and Precocious Puberty: A Case Series and Literature Review

**DOI:** 10.3389/fendo.2021.672394

**Published:** 2021-10-29

**Authors:** Xiao Zhai, Lian Duan, Yong Yao, Bing Xing, Kan Deng, Linjie Wang, Feng Feng, Zhiyong Liang, Hui You, Hongbo Yang, Lin Lu, Shi Chen, Renzhi Wang, Hui Pan, Huijuan Zhu

**Affiliations:** ^1^Key Laboratory of Endocrinology of National Health Commission, Department of Endocrinology, State Key Laboratory of Complex Severe and Rare Diseases Peking Union Medical College Hospital, Chinese Academy of Medical Science and Peking Union Medical College, Beijing, China; ^2^Department of Endocrinology, Peking Union Medical College Hospital, Chinese Academy of Medical Science (CAMS), Beijing, China; ^3^Department of Neurosurgery, Peking Union Medical College Hospital, Chinese Academy of Medical Science and Peking Union Medical College, Beijing, China; ^4^Department of Radiology, Peking Union Medical College Hospital, Chinese Academy of Medical Science and Peking Union Medical College, Beijing, China; ^5^Department of Pathology, Peking Union Medical College Hospital, Chinese Academy of Medical Science and Peking Union Medical College, Beijing, China

**Keywords:** McCune-Albright syndrome, GH excess, precocious puberty (PP), gigantism, acromegaly

## Abstract

**Background:**

McCune-Albright syndrome is a rare disorder characterized by fibrous dysplasia, café au lait skin spots, and hyperfunctioning endocrinopathies. The coexistence of precocious puberty and growth hormone excess in McCune-Albright syndrome is rare. Both conditions can manifest as accelerated growth, and treatments can be more challenging for such patients. This study aimed to describe the clinical manifestations of combined GH excess and PP in the context of McCune-Albright syndrome and analyze the clinical features and treatments of these patients.

**Method:**

Clinical data from 60 McCune-Albright syndrome patients from Peking Union Medical College Hospital were obtained. The demographic characteristics, growth hormone, insulin-like growth factor-1, prolactin, alkaline phosphatase, and sex hormone levels; growth velocity; and bone age data were obtained. The growth velocity Z-score, bone age over chronological age ratio, and predicted adult height Z-score were calculated before and after treatment. Published studies and case reports were systemically searched, and data on demographic, clinical, and biochemical characteristics and treatment outcomes were obtained.

**Results:**

We reviewed seven patients among 60 McCune-Albright syndrome patients at Peking Union Medical College Hospital (5 female) and 39 patients (25 female) from the published literature. Six of the seven patients from Peking Union Medical College Hospital and half of the patients from the published studies were pediatric patients. These patients had increased growth velocity Z-scores and bone age over chronological age ratios. After good control of both conditions, the growth velocity Z-score and bone age over chronological age ratio decreased significantly, and the predicted adult height Z-score increased. The final heights and predicted adult height Z-scores were not impaired in patients with gigantism. All the patients had craniofacial fibrous dysplasia associated with optic and otologic complications.

**Conclusion:**

McCune-Albright syndrome with growth hormone excess and precocious puberty is more common in girls. Patients have accelerated linear growth and advanced skeletal age, and early and good control of both conditions leads to a reduced growth velocity and stabilized bone age. The predicted adult and final heights are not negatively affected when growth hormone excess is diagnosed in pediatric patients.

## Background

McCune-Albright syndrome (MAS) is a sporadic disease caused by somatic activating mutations of the *GNAS1* gene encoding the α subunit of guanine nucleotide-binding protein (1), which lead to constitutive receptor activation and dysregulated production of cAMP (2). MAS is characterized by the triad of monostotic/polyostotic fibrous dysplasia (FD), café au lait skin pigmentation, and hyperfunctioning endocrinopathies, including gonadotropin-independent precocious puberty (PP), thyrotoxicosis, growth hormone (GH) excess, hyperprolactinemia, or neonatal hypercortisolism (2–6).

MAS is a rare disease, and the estimated prevalence is between 1/100,000 and 1/1,000,000 (7). Gonadotropin-independent sexual puberty is the most common endocrinopathy in MAS (4), affecting 50% of girls, and is far more common in girls than in boys with MAS (8). MAS-associated precocious puberty (PP) is a rare cause of PP, and the estimated prevalence of PP (including gonadotropin-dependent and gonadotropin-independent PP) in the general population from a Danish national registry was 0.2% in female patients and less than 0.05% in males (9). Progression to gonadotropin-dependent PP over time has also been documented in some patients (10, 11). MAS-associated PP in girls is caused by recurrent, unilateral autonomously functioning ovarian cysts, which leads to episodic estrogen production with suppressed gonadotropins (12). Girls typically present with breast development and painless vaginal bleeding. PP in boys with MAS is associated with a premature penile growth and bilateral testicular enlargement, secondary to Leydig cell hyperplasia and elevated testosterone production (5). Additionally, PP causes growth acceleration and skeletal advancement (13), leading to impaired adult height. GH excess, which is present in 20~30% of patients with MAS (8, 14–16), presents with increased growth velocity in children and adolescents, and facial and acral dysmorphia in adults. GH excess is usually accompanied by serious craniofacial FD complications—e.g., visual, hearing, and olfactory injuries (16–22).

The co-occurrence of PP and GH excess in MAS is rare. To date, only a few case reports regarding GH excess and PP in MAS have been reported. The diagnosis and treatment of GH excess may become challenging when PP is also present. GH excess and PP both present with accelerated linear growth, and facial dysmorphia is often difficult to assess because of craniofacial fibrous dysplasia (CFFD). Therefore, it is easy to miss the diagnosis of GH excess because of the coexistence of PP and CFFD. Additionally, choosing the appropriate treatment of GH excess and PP is essential for children and adolescents to achieve normal adult height.

This study aimed to retrospectively analyze the clinical manifestations, treatments, and outcomes of combined GH excess and PP in MAS patients from Peking Union Medical College Hospital (PUMCH), review all reported cases, and analyze the clinical features and treatments of these patients.

## Subjects and Methods

### Patients

**Figure 1:**
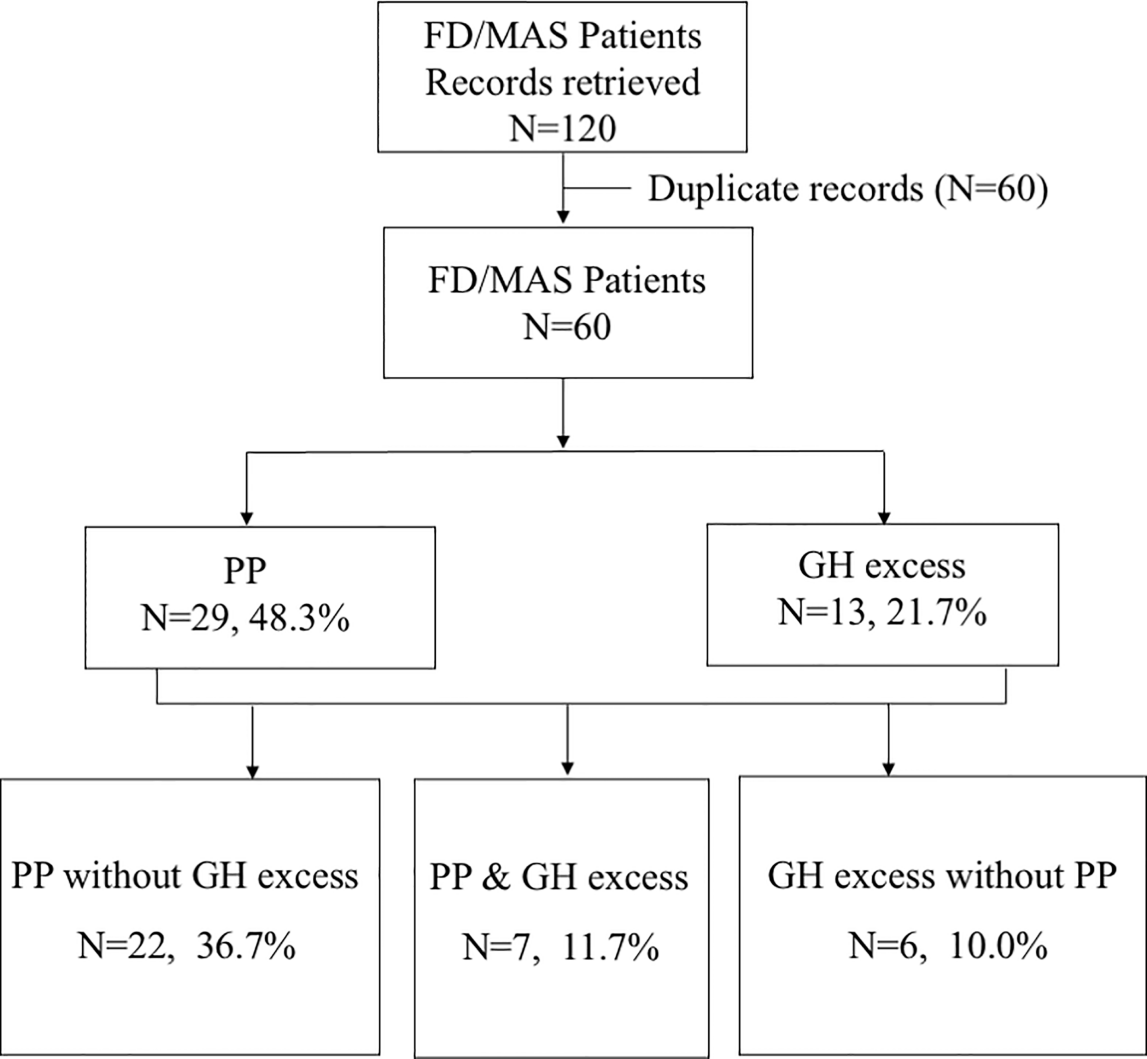
Flowchart of the study population.

This study was conducted in accordance with the rules of the hospital medical ethics committee, and informed consent was obtained. A retrospective study was performed on seven MAS patients who had a combined diagnosis of GH excess (including gigantism and acromegaly) and PP over 10 years (2010~2020) at PUMCH. The inclusion and exclusion process is shown in **Figure 1**.

### Literature Review

All the studies and case reports of GH excess and PP with MAS (GH excess, acromegaly, gigantism, precocious puberty, and McCune-Albright syndrome were the keywords) were systematically sought in the world literature up to September 2020. Studies and case reports were identified in the PubMed, Embase, Google Scholar, and Chinese Biomedical (CBM) databases, and those with an English abstract were included in the analysis.

### Data Extraction

We extracted data from medical records in PUMCH and published studies and analyzed each patient in case reports and case series. Pretreatment data were routinely collected before the initiation of treatment, and posttreatment data were collected at the last consultation for patients under therapy. The following data were extracted (1): demographic characteristics (2); age at MAS, GH excess, and PP diagnosis (3); type of fibrous dysplasia: craniofacial fibrous dysplasia (CFFD) *versus* polyostotic FD (4); pituitary imaging findings (5); GH, insulin-like growth factor (IGF-1), and prolactin (PRL) levels, bone age (BA) before and after treatment (6); alkaline phosphatase (ALP) levels before and after treatment (7); transabdominal pelvic (females) or testicular ultrasounds (males) (ovarian and testicular volumes were calculated using the formula volume = length × width × thickness × 0.52) (8); treatment for GH excess (9); treatment for PP; and (10) final heights and his/her parents’ heights.

### Diagnosis and Treatment of MAS, GH Excess, and PP

A diagnosis of MAS was made when at least two of the following major features existed: fibrous dysplasia of bone, café au lait skin pigmentation, and hyperfunctioning endocrinopathies.

The diagnosis of GH excess was based on the presence of typical clinical manifestations (accelerated linear growth, facial dysmorphia, shoe size modification, visual defects, and/or headaches), elevated IGF-1 levels (as assessed using the IMMULITE 2000 IGF-1 analyzer; Siemens Healthcare Diagnostic Inc.), and GH nadir ≥1 µg/L following documented hyperglycemia during an oral glucose load. The IGF-1 Z-scores were calculated according to the normal values of serum IGF-1 (5th and 95th percentiles) with adjustment for age and sex (15, 23, 24), and Z-scores greater than 2.0 were considered elevated. Gigantism is defined when GH excess leads to linear growth acceleration before the end of puberty and epiphyseal closure, while acromegaly is determined when GH excess is present in individuals after epiphyseal closure. Complete remission of GH excess is defined as a normal IGF-1 level for age and sex (Z score<2.0), and GH nadir<1 µg/L after the oral glucose tolerance test (OGTT).

Gonadotropin-independent PP is defined as the onset of secondary sexual characteristics before the age of 8 years in girls and 9 years in boys (25), with excess secretion of sex hormones (estrogens or androgens) and suppressed production of gonadotropins. The gonadotropin-releasing hormone (GnRH) stimulation test was performed to exclude gonadotropin-dependent PP. Girls typically present with painless vaginal bleeding with breast development in early childhood, and boys present with a premature increase in penile size and mild bilateral testicular enlargement (13).

### Height and Skeletal Measurements

Height measurements were taken at PUMCH using a stadiometer and reported as the average of three consecutive morning values. BAs were determined using the Greulich and Pyle atlas reading method (26). The predicted adult height (PAH) was calculated according to the Bayley-Pinneau method (27). The baseline BA and height measurements were obtained before the initiation of treatment. Height and growth velocity (GV) Z-scores were determined based on normative reference data for children and adolescents in China (28, 29). Height Z-scores from the literature review were based on normative reference data for children and adolescents of the World Health Organization (30). Posttreatment data were obtained at the time of treatment discontinuation or last evaluation for those remaining on treatment. Additionally, the predicted height was calculated as the mean parental height plus 6.5 cm for male patients and minus 6.5 cm for female patients. The ALP Z-scores were based on the reported distributions of ALP levels in the Chinese population (31).

### Statistical Analysis

Statistical analyses were performed using SPSS 23.0, and figures were prepared using GraphPad Prism, version 6 (GraphPad Software Inc.). The data are presented as the means (minimal, max) or medians (minimal, max) as appropriate depending on the normality of the distribution. Paired samples t-tests were performed to compare patients before and after treatment. Independent sample t-tests were performed to make comparisons between GH excess patients diagnosed with MAS with or without PP. P values <0.05 were considered statistically significant.

## Results

### Clinical Characteristics and Treatment Outcomes in PUMCH

#### Baseline Demographic and Clinical Features

Seven MAS patients from PUMCH who had been diagnosed with GH excess and PP were included in this study; five were female (71.4%), and the mean age was 5.4 years (min,max 2.1, 8.9 years) of the diagnosis of MAS (**Table 1**). Café au lait spots were present in five individuals (71.4%). The mean age of the individuals at the initial development of signs of PP was 4.3 years (1.0, 7.0 years). In girls, the symptoms prompting evaluation were vaginal bleeding in three subjects (patients 1, 4, and 7) and breast development in two subjects (patients 3 and 5). Pelvic ultrasound showed unilateral (patients 1, 5, and 7) or bilateral (patient 3) ovarian cysts. In boys, the diagnosis was suggested by acne, scrotum enlargement, and erection. Testicular ultrasound showed bilateral testicular enlargement in patients 2 and 6 and focal hyperechoic lesions in patient 6. Subjects showed advanced skeletal maturation [median BA over chronological age (BA/CA) ratio: 1.44; 1.39, 1.98]. Six of the seven patients had gigantism, and the mean age at diagnosis was 6.5 years (4.7, 8.9 years). The presenting sign of the patients was accelerated linear growth. Patient 4 had experienced intermittent vaginal bleeding and focal bone dysplasia since the age of 7 years, and she was diagnosed with acromegaly at the age of 27 years in routine assessment during follow-up for MAS. CFFD was present in all the patients from PUMCH, among whom three patients (42.9%) had visual field deficits, two patients (28.6%) had conductive hearing loss, and one patient (14.3%) had olfactory dysfunction. The clinical characteristics of the patients from PUMCH are listed in **Table 1**.

**Table 1:** Demographic and clinical characteristics of PUMCH patients.

Patient ID	Sex	Age at dx of MAS (y)	Age at sx of PP (y)	Age at dx ofGH excess (y)	Café au lait	Polyostotic FD	CFFD	Height Z-score	Other endocrinopathies	Hearing or olfactory deficits	Visual Deficits
1	F	6.3	5.0	6.3	+	+	+	3.3	None	Unilateral hyposmia	Bilateral temporal hemianopia
2	M	8.9	7.0	8.9	+	+	+	2.1	None	Hyposmia	Unilateral visual field defect
3	F	4.7	1.0	4.7	+	+	+	4.0	None	None	None
4	F	7.0	7.0	27	−	+	+	-0.7	None	None	Diplopia
5	F	4.3	4.0	6.6	−	+	+	4.1	None	CHL	None
6	M	6.3	5.0	6.3	+	+	+	4.53	None	CHL	Unilateral visual field defect
7	F	2.1	1.0	6.4	+	+	+	1.9	Hyperthyroidism	None	None

CHL, conductive hearing loss; Dx, diagnosis; Sx, symptom and sign; FD, fibrous dysplasia; CFFD, craniofacial fibrous dysplasia; PP, precocious puberty. Height Z-score was recorded at the first visit.

#### Hormone Measurements and Imaging Findings

**Figure 2:**
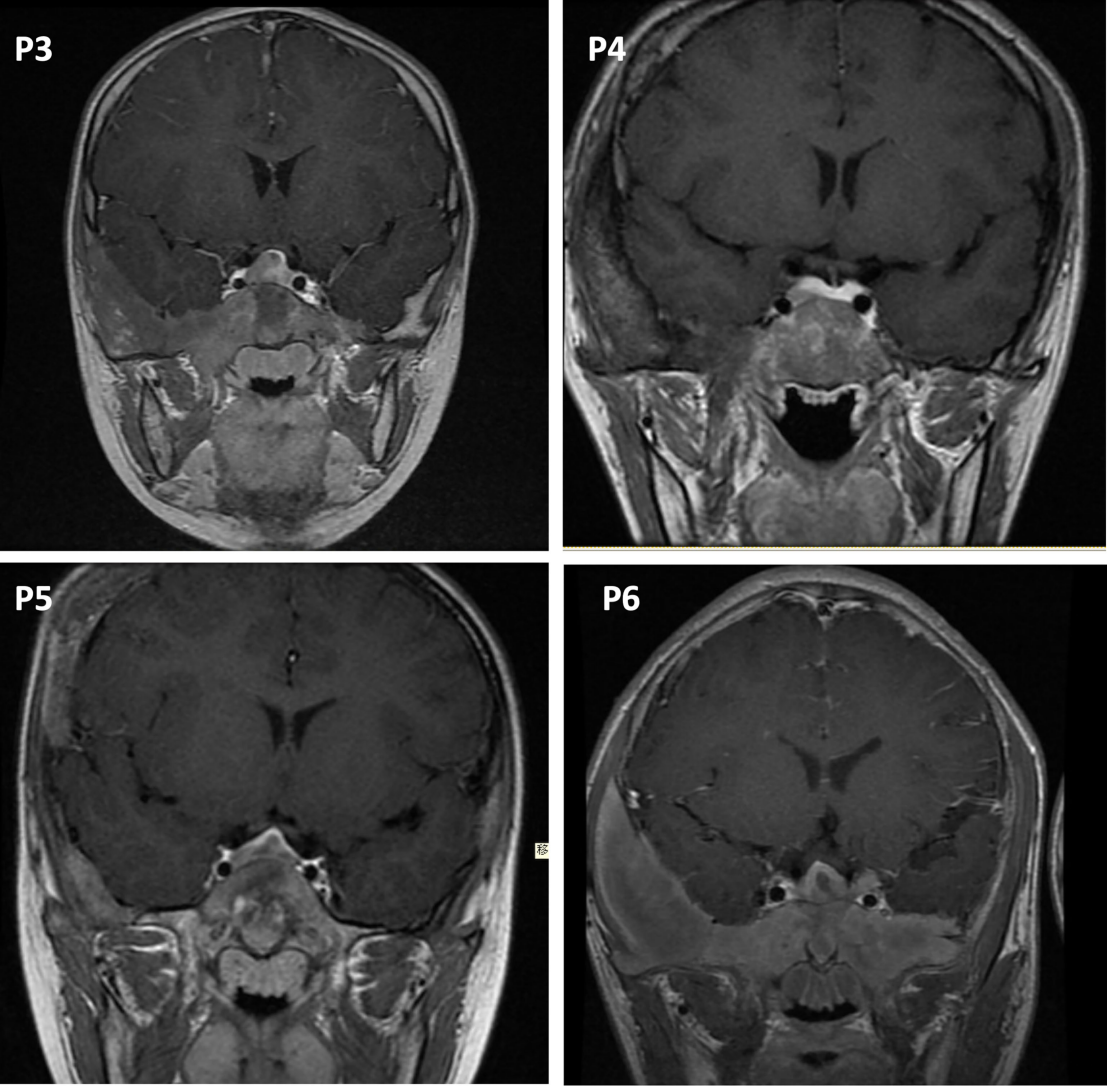
Pituitary MRI in the coronal view. P3, patient 3, macroadenoma. P4, patient 4, microadenoma. P5, patient 5, multiple microadenomas. P6, patient 6, macroadenoma with a cystic zone.

**Figure 3:**
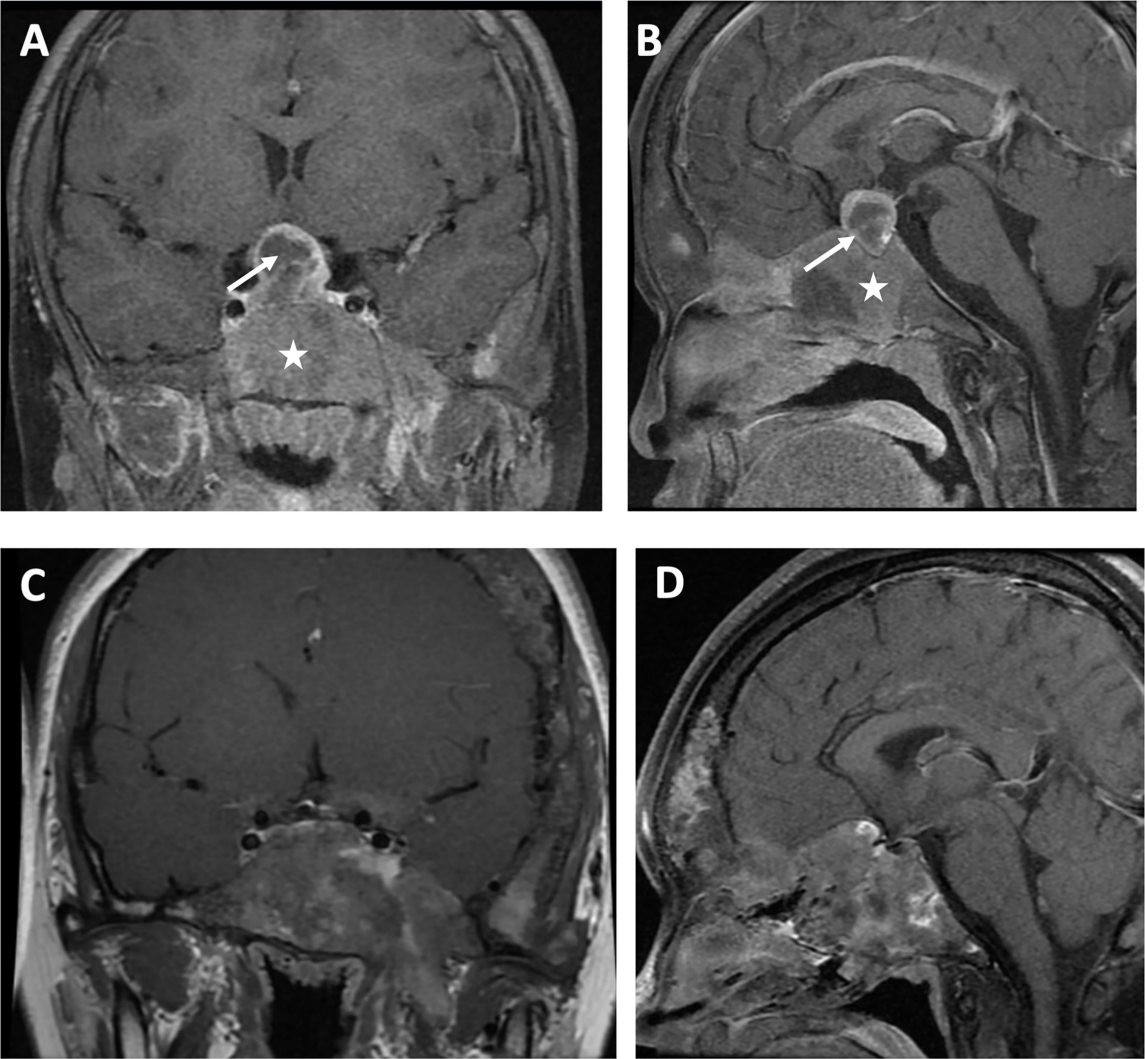
Visualization by MRI in the coronal view **(A)** and sagittal view **(B)** of a macroadenoma (arrow) surrounded by cranial fibrous dysplasia (star) in a patient with MAS and GH excess before surgery and pharmacologic treatment. Coronal view **(C)** and sagittal view **(D)** after surgery and pharmacologic treatment.

For hormone measurements, the mean random GH level, GH nadir after OGTT, and IGF-1 Z-score at the diagnosis of GH excess at PUMCH were 14.1 µg/L (4.9, 33.6), 13.2 µg/L (5.2, 35.3), and 6.4 (2.0, 10.2), respectively. Hyperprolactinemia was present in four patients (57.1%, mean 70.5 ng/ml; 47.7, 204.4), and a value greater than 200 ng/ml was only observed in patient 1. Magnetic resonance imaging (MRI) data were available for six individuals (**Figure 2**), revealing three macroadenomas and one microadenoma; patient 5 had multiple microadenomas, and patient 7 had normal MRI imaging findings. MRI imaging of patient 1 before and after surgery and pharmacological treatment is illustrated in **Figure 3**. Regarding other endocrinopathies, hyperthyroidism was present in one patient from PUMCH who had endocrinopathies accompanied by GH excess and PP in MAS (1/7; 14.3%).

#### Treatment of PP and GH Excess

The treatment for PP varied at PUMCH (the clinical characteristics and treatment outcomes of PP at PUMCH are listed in **Table 2**). Letrozole was used in two of seven patients, either alone (patient 6) or in combination with medroxyprogesterone (patient 7). Patient 5 was treated with tamoxifen. Patient 2 was treated with a GnRH agonist (GnRHa) because of central PP secondary to peripheral PP. However, after 3 months of treatment, patient 2 discontinued GnRHa because of financial restriction. Patients 1 and 3 were closely monitored without medical treatment. Their symptoms were relatively stable, and their BA/CA ratios were close to the normal range.

**Table 2:** Clinical and biochemical characteristics and treatment outcomes of patients with PP from PUMCH.

Patient ID		Pretreatment								Tx for PP	Tx duration (y)			Posttreatment								
	BA/CA	PAHZ-score	GV (cm/y)Z-score	LH (IU/L)	Peak LH(IU/L)	FSH (IU/L)	E2 (pg/ml)	T(ng/ml)	Mean testicular or ovarian volumn(mm^3^)			BA/CA	PAHZ-score	Predicted Ht (cm)	Predicted HtZ-score	Final HtZ-score	GV (cm/y)Z-score	LH (IU/L)	FSH(IU/L)	E2(pg/ml)	T (ng/ml)	Mean testicular or ovarian volumn(mm^3^)
1	1.44	+4.9	+8.99	0.21	2.2	2.10	10.2	<0.1	1.6	None		1.0		157.5	−0.5	+3.3	−2.10					
2	1.43	−0.2	NA	2.60	25.6	8.40	21.1	1.39	4.2	GnRHa	0.25	NA	NA	171.0	−0.2	−0.3	NA	0.40	0.46	26.30	0.21	NA
3	1.39	+2.1	+12.40	<0.2	1.15	0.25	216.0	<0.1	1.8	None		1.02	+2.6	161.5	0.1		−0.6	<0.2	0.25	11.05	<0.1	2.6
5	1.53	+0.41	+13.38	<0.2	0.98	0.78	165.0	0.31	7.5	Tamoxifen 5 mg bid	5.25	1.24	+2.1	162.5	0.3		+1.91	<0.2	0.31	33.00	0.22	1.7
6	1.98	−0.3	+11.72	1.14	1.20	0.78	21.0	1.65	3.3	Letrozole 2.5 mg qod Medroxyprogesterone 4 mg bid	2.25	1.53	+2.1	174.5	0.3		−0.94	<0.2	0.32	<5	1.12	4.4
7	1.40	+0.2	+10.97	<0.2	3.76	0.11	57.73	<0.1	2.3	Letrozole 1.25 mg qd	0.5	1.27	+0.9	163	0.4		2.35	0.26	3.36	11.06	<0.1	1.3

BA/CA, bone age over chronological age; PAH, predicted adult height; GV, growth velocity; LH, luteinizing hormone; FSH, follicle-stimulating hormone; Tx, treatment; E2, estradiol; T, testosterone; Y, year; Ht, height; NA, not available. Peak LH was LH level after GnRH stimulation test. The posttreatment sex hormones and average ovarian measurements for patient 1 are not listed because she was sexually mature at the last follow-up at age 17.

**Figure 4:**
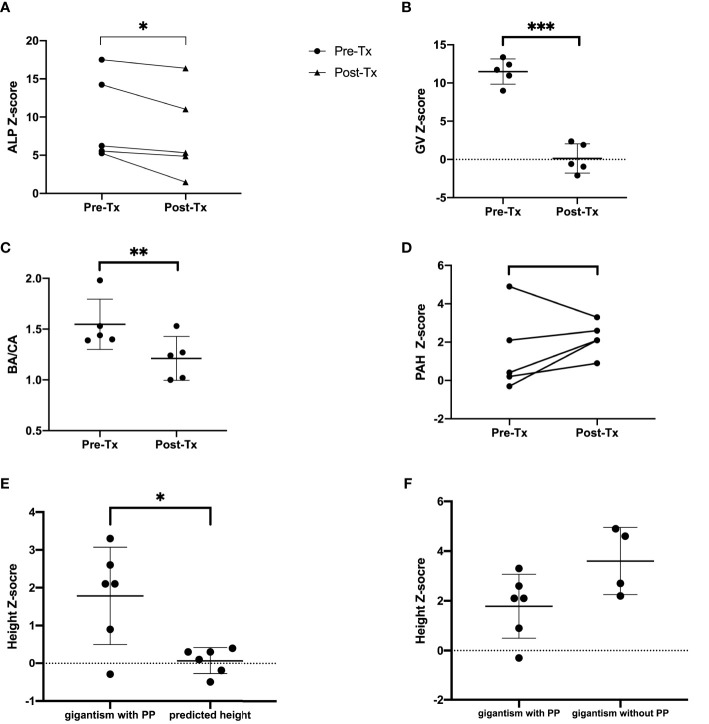
ALP-Z score **(A)**, GV Z-score **(B)**, BA/CA ratio **(C)**, PAH Z-score **(D)** before and after treatment. The post-Tx data were collected at the last consultation for patients under therapy. **(E)** Final height or PAH Z-scores of patients diagnosed with gigantism and PP of MAS *versus* their predicted adult height. **(F)** Final height Z-scores of patients diagnosed with gigantism and PP *versus* final height or PAH Z-scores of patients diagnosed with gigantism without PP. Tx, treatment; Pre-Tx, pretreatment; Post-Tx,posttreatment; ALP, alkaline phosphatase; GV, growth velocity; BA/CA, bone age over chronological age; PAH, predicted adult height. ***P < 0.001, **P < 0.01, *P < 0.05.

Five patients (71.4%) from PUMCH had undergone navigation-assisted transsphenoidal pituitary tumor resection, and two patients achieved complete remission (the clinical characteristics and treatment outcomes of patients with GH excess are listed in **Table 3**). Three patients (patients 1, 3, and 6) had partial remission after surgery and continued pharmacological treatment. Patient 1 then received treatment with dopamine agonist (DA) bromocriptine and long-acting somatostatin analog (LAR) octreotide (Sandostatin LAR, Novartis). The random GH level fell to 3.7 ng/ml, and the IGF-1 level was 601 ng/ml (IGF-1 Z-score 0.3). LAR was also effective in further lowering the IGF-1 levels after surgery in patient 3, but she had a partial response. Patient 6 was treated with bromocriptine alone after surgery, and the patient’s GH excess was partially controlled. Patient 7 was closely followed up without surgical or pharmacological treatment because her IGF-1 Z-score was slightly elevated and her GV was within the normal range. Visual and auditory complications remained stable after treatment. The ALP Z-score decreased significantly after successful treatment with GH excess and PP (*P*=0.04, **Figure 4A**).

**Table 3:** Clinical and biochemical characteristics and treatment outcomes of patients with GH excess and PP from PUMCH.

Patient ID	Pretreatment						Posttreatment					Outcome
	GH nadir(µg/L)	IGF-1Z-score	PRL(µg/L)	ALPZ-score	MRI (largest diameter; mm)	Treatment for GH excess	IHC postivity	GH nadir(µg/L)	IGF-1Z-score	PRL(µg/L)	ALPZ-score	
1	35.3	10.2	204.4	+5.25	Macro (24)	Surgery+LAR+DA	GH,PRL	3.1	0.3	24.4	+1.48	PR
2	8.3	7.8	21.0	+9.97	NA	None		–	–	–	–	NA
3	NA	10.0	143.4	+5.54	Macro (14)	LAR + Surgery	GH,PRL	2.52	2.64	21.1	+4.85	PR
4	5.9	2.2	13.7	+17.50	Micro (7)	Surgery	GH	0.1	−0.6	7.8	+16.39	CR
5	5.2	6.9	51.8	+6.22	Multiple micro	Surgery	GH	0.748	−0.9	22.1	+5.32	CR
6	21.4	5.7	47.7	+14.24	Macro (19)	Surgery+DA	GH	6.4	5.0	4.7	+11.02	PR
7	3.0	2.0	11.19	+21.28	Absence	None		–	–	–	–	–

GH, growth hormone; IGF-1, insulin-like growth factor-1; PRL, prolactin; ALP, alkaline phosphatase; Macro, macroadenoma; Micro, microadenoma; LAR, long-acting somatostatin analogue octreotide; DA, dopamine agonist; NA, not available. The reference value for GH nadir is less than 1 µg/L. The reference value of prolactin for females is less than 30 µg/L in female patients, and 2.6~13.1 µg/L in male patients.

#### Growth Outcome

For patients diagnosed before the age of 16 years at PUMCH (patients 1, 3, 5, 6, and 7 were included; patient 2 was not included in the analysis because of a relatively short follow-up time), there was accelerated linear growth (mean GV Z-score: 11.5; 9.0, 13.4) and overall advanced skeletal maturation (mean BA/CA ratio: 1.53; 1.39, 1.98). After good control of GH excess and PP, the mean GV Z-score decreased from 11.49 to 0.12, which was statistically significant (*P <*0.001) (**Figure 4B**). The median BA/CA ratio also decreased significantly (1.44 to 1.24; *P* =0.005) (**Figure 4C**). The posttreatment PAH or final height Z-score (mean PAH or final height Z-score 1.8) was higher than the predicted height Z score (mean predicted height Z-score 0.1; *P*=0.03) (**Figure 4E**). The PAH Z-scores were normal (−2 to +2) in four of six patients (66.7%; patients 2, 5, 6, and 7), and patients 1 and 3 had increased PAH Z-scores. Most of the patients had PAH Z-scores that increased after treatment except for patient 1. The mean PAH Z-score increased from 1.46 to 2.2 after treatment, with no statistical significance (**Figure 4D**). The GV Z-score of patient 1 decreased from 8.99 to −2.1 after surgical and pharmacological treatment. Patient 4, diagnosed with acromegaly at the age of 27, had a final height of 156 cm (Z-score −0.7) and a predicted height of 159 cm (Z-score −0.3).

Six patients were diagnosed with GH excess without PP in our cohort (the clinical characteristics and final height of these patients are illustrated in **Table 4**), four of six had symptoms of gigantism before the age of 16 (without a diagnosis or treatment until adulthood), and two of six were diagnosed in adulthood. The final height Z-score of gigantic patients without PP (mean final height Z-score: 3.6; 2.2, 4.9) was higher than the final height Z-score or posttreatment PAH Z-score of patients codiagnosed with gigantism and PP (mean Z-score: 1.8; −0.3, 3.3; *P*=0.064) (**Figure 4F**).

**Table 4:** Clinical characteristics and final height of patients diagnosed with GH excess without PP from PUMCH.

Patient ID	Sex	Café au lait	Age of Dx of MAS (y)	Age of Sx of GH excess (y)	CFFD	Polyostotic FD	Final Ht(cm)	Final Ht Z-score	Predicated Ht(cm)	Predicated HtZ-score
1	M	Y	26	15	Y	Y	188	2.2	179	0.9
2	M	Y	9	9	Y	Y	204	4.6	NA	NA
3	F	N	24	10	Y	Y	191f	4.9	164.5	0.6
4	M	Y	34	15	Y	Y	191.3	2.7	181.5	1.3
5	M	Y	11	45	Y	Y	173	0.0	NA	NA
6	M	Y	16	23	Y	Y	183.5	1.6	181.5	1.3

Dx, diagnosis; Sx, symptom and sign; FD, fibrous dysplasia; CFFD, craniofacial fibrous dysplasia; Ht, height; NA, not available.

### Clinical Characteristics and Treatment Outcomes From the Literature Review

The clinical characteristics, treatment, and outcomes of patients with MAS with GH excess and PP from previous case series and reports are summarized in **Tables 5** and **6**. Thirty-nine (25 females, 64.1%) patients were analyzed. Regarding GH excess, 13 patients were diagnosed before the age of 16 years old (mean age: 7.7 years; 4, 13), and 13 patients (50%) were diagnosed in adulthood, with an average age of 27.1 years (17.8, 38). The mean random GH level and IGF-1 Z-score were 39.0 µg/L (2.8, 290) and 5.9 (−0.2, 17.7), respectively. MRI data were available for 27 of 39 cases (69.2%), and CT and X-ray scans were performed for eight patients, revealing 14 macroadenomas (51.9%) and 7 (25.9%) microadenomas. For craniofacial FD complications, 11 patients had various degrees of visual impairment, and five patients had olfactory impairment. Five patients received treatment for PP among the 19 patients with available data. Aromatase inhibitors, tamoxifen, GnRHas, testolactone, anastrozole, and medroxyprogesterone acetate were administered (cases 3, 15, 16, 27, and 34). Syptoms of PP were well controlled in cases 15 and 16, and partially controlled in case 34. The response to treatment of PP was not mentioned in case 3 or 27. Eight (36.4%) out of 22 patients for whom data were available achieved complete remission, 10 (45.5%) individuals had partial remission, GH excess was not controlled in 4 (18.2%) individuals, and 1 (4.5%) patient died after surgery from postoperative hemorrhage. Among the patients with complete disease resolution, one patient was cured by surgery; one patient was treated with a DA alone; one patient was cured by an LAR and DA; and the remaining five patients received combination treatment with either pegvisomant, an LAR and DA or pegvisomant and fractioned radiotherapy.

**Table 5:** Clinical characteristics of patients from the literature review.

Patient No.	First author, year	Type of study	Sex	Age at Dx (y)	Age at dx of GH excess (y)	Age at dx of PP (y)	CAL	CFFD	Polyostotic FD	Other endocrinopathies	Imaging	Type of lesion on imaging (largest diameter; mm)	Treatment for PP	Ovarian or testicular ultrasound
1	Rajan, 2019 (32)	Case report	F	24	24	NA, menarche at 7	+	+	+	Hyperthyroidism	MRI	Macro (40)	None	NA
2	Franco, 2019 (33)	Case report	F	26	26	9	+	+	+		MRI	Macro	None	NA
3	Wong, 2017 (34)	Cohort study	M	2	8	NA	+	+	+	Hyperthyroidism	MRI	Absence	Aromatase inhibitor and tamoxifen, GnRHa	Heterogeneous changes Echogenic lesions Microlithiasis
4	Wong, 2017 (34)	Cohort study	M	3	NA	6	+	+	+	Hyperthyroidism	MRI	Micro (4)	None	Echogenic lesions Microlithiasis
5	Wong, 2017 (34)	Cohort study	F	36	36	NA	+	+	+	Hyperthyroidism	MRI	Adenoma	None	NA
6	Akintoye, 2006 (35)	Randomized controlled crossover study	F	13	NA	NA	+	+	+	Hyperthyroidism	MRI	Abnormal enhancement	NA	NA
7	Vortmeyer, 2012 (36)	Case series	F	29	NA	NA	+	+	+		MRI	Micro	NA	NA
8	Vortmeyer, 2012 (36)	Case series	M	19	NA	NA	+	+	+		MRI	Macro	NA	NA
9	Classen, 2012 (37)	Case report	F	3	11	NA	−	+	NA		MRI	Micro	None	NA
10	Nozieres, 2011 (38)	Cohort study	M	6.5	6	NA	+	+	+		MRI	Hyperplasia	NA	NA
11	Madsen, 2010 (39)	Case series	F	8.2	8.2	5	−	+	+		MRI	Micro	None	NA
12	Almeida, 2009 (40)	Case report	F	29	29	NA	+	+	+		MRI	Macro (19)	None	NA
13	Imanaka, 2007 (41)	Case report	F	5	21	4	+	+	−	Hyperthyroidism	MRI	Macro (15)	NA	NA
14	Galland, 2006 (42)	Cohort study	F	27	27	NA	+	+	+	Hyperthyroidism	MRI	Macro	NA	NA
15	Papadopoulou, 2006 (43)	Case report	M	9	9	9	+	+	+		MRI	Micro (9)	testolactone	Microlithiasis
16	Zacharin, 2005 (44)	Case report	M	2.5	8.5	5	+	+	+		MRI	Hyperplasia	Testolactone→anastrozole	NA
17	Akintoye, 2002 (15)	Cohort study	M	7	NA	NA	+	+	+	Hyperthyroidism	MRI	Abnormal enhancement	NA	NA
18	Akintoye, 2002 (15)	Cohort study	M	30	NA	NA	+	+	+		MRI	Macro (17)	NA	NA
19	Akintoye, 2002 (15)	Cohort study	F	34	NA	NA	+	+	+		MRI	Macro (17)	NA	NA
20	Akintoye, 2002 (15)	Cohort study	F	5	NA	NA	+	+	+	Hyperthyroidism	MRI	Absence	NA	NA
21	Akintoye, 2002 (15)	Cohort study	F	26	NA	NA	+	+	+		MRI	Micro (9)	NA	NA
22	Akintoye, 2002 (15)	Cohort study	F	11	NA	NA	+	+	+	Hyperthyroidism	MRI	Absence	NA	NA
23	Akintoye, 2002 (15)	Cohort study	F	4	NA	NA	+	+	+		MRI	Absence	NA	NA
24	Akintoye, 2002 (15)	Cohort study	F	13	NA	NA	+	+	+	Hyperthyroidism	MRI	Adenoma	NA	NA
25	Zumkeller, 2001 (45)	Case report	M	8	8	7	+	+	+	Hyperthyroidism	MRI	Micro (4)	NA	NA
26	Dotsch, 1996 (46)	Case report	M	6.5	6.5	6.5	−	+	+		MRI	Macro (18)	NA	NA
27	Feuillan, 1995 (47)	Case report	F	6 months	7.3	6 months	+	+	+	Hyperthyroidism	MRI	Absence	Testolactone	NA
28	Premawardhana, 1992 (48)	Case report	F	26	26	3	+	+	+	Adrenal insufficiency	CT	Macro	None	NA
29	Abs, 1990 (49)	Case report	F	36	36	8	−	+	+	Hyperthyroidism	MRI	Macro (15)	None	NA
30	Laughlin, 1989 (50)	Case report	F	9	13	9	+	+	+		MRI	Macro (36)	NA	NA
31	Cuttler, 1989 (51)	Case series	M	9.5	NA	NA	+	+	+		NA	NA	None	NA
32	Geffner, 1987 (52)	Case report	F	8 months	17.8	8 months	+	+	+	Hyperthyroidism	CT	Macro (21)	none	NA
33	Mauras, 1986 (53)	Case report	M	4	4	NA	+	+	+	Hyperthyroidism, CS	CT	NA	NA	NA
34	Kovacs, 1984 (54)	Case report	F	4	6	4	+	+	+		CT	NA	Medroxyprogesterone acetate	NA
35	Albin, 1981 (55)	Case series	F	19	19	NA	+	+	+		XR	NA	None	NA
36	Albin, 1981 (55)	Case series	M	23	23	NA	+	+	+		XR	Absence	None	NA
37	Lightner, 1975 (56)	Case report	M	4	4	4	+	+	+		XR	Macro (15)	NA	NA
38	Scurry, 1964 (57)	Case report	F	22	38	5	+	+	+		NA	NA	NA	NA
39	Carr, 1979 (58)	Case report	F	30	30	19 months	+	+	+		XR		None	NA

Dx, diagnosis; FD, fibrous dysplasia; CFFD, craniofacial fibrous dysplasia; PP, precocious puberty; GH, growth hormone; CAL, Café au lait; CS, Cushing’s syndrome. The number in brackets of type of lesion is the largest diameter of the pituitary tumor. NA, not available.

**Table 6:** Biochemical characteristics and treatment outcomes of patients with GH excess from the literature review.

Patient No.	Pretreatment				Tx for GH excess	Posttreatment			Visual defect	Hearing and olfactory defects
	GH nadir(µg/L)	IGF-1Z-score	PRL(µg/L)	ALP(IU/L)		IGF-1Z-score	ALP(IU/L)	GH excesscontrolled		
1	8.21	8.1	3,218	216	DA	NA	NA	NA	Bitemporal hemianopia	NA
2	NA	14.8	155.8	2,259	LAR+PEG	NA	NA	NA	Left eye dystopia	NA
3	NA	2.0	24.9	NA	LAR+DA	NA	NA	No	NA	NA
4	NA	2.0	20	NA	LAR+DA	NA	NA	Yes	NA	NA
5	NA	4.5	4.43	NA	LAR+DA	NA	NA	No	NA	NA
6	NA	1.8	NA	715	LAR+DA+PEG	−2.8	515	Yes	NA	NA
7	40.5	6.2	26	NA	Surgery	1	NA	PR	Unilateral blindness	Deafness
8	127	8.8	17	NA	Surgery	−2.4	NA	Yes	NA	NA
9	NA	6.0	285.3	690	DA	NA	417	Yes	Mild left-sided hemianopia	NA
10	NA	3.0	NA	NA	LAR+DA+PEG	−1	NA	Yes	NA	NA
11	NA	17.7	38.5	NA	2 surgeries + LAR + DA	3.6	NA	PR	NA	NA
12	3.4	3.0	177	537.9	Surgery + LAR + DA	2.3	NA	PR	NA	NA
13	NA	2.3	18.9	8721	LAR	−0.3	6,870	PR	Bitemporal upper quarter blindness	NA
14	NA	8.3	18	NA	RT+PEG	0.7	NA	Yes	NA	NA
15	12.5	NA	NA	NA	LAR	NA	NA	NA	Normal	Normal
16	26	NA	NA	812	LAR		NA	PR	Binasal visual field loss	NA
17	1.2	1.8	20	1224	LAR+DA+PEG	−2.5	970	Yes	Normal	Normal
18	60.2	8.2	81.5	474	LAR+DA/PEG	3	366	PR	Blindness	Hearing loss
19	14.3	4.6	98	871	LAR+DA/PEG	1.1	833	Yes	Normal	Normal
20	16.2	3.2	27	NA	LAR	NA	NA	NA	Normal	Normal
21	29	>5	53	NA	DA	NA	NA	NA	Normal	Normal
22	2.3	−0.2	36	NA	None	NA	NA	NA	Normal	Normal
23	5.3	2.5	17	NA	LAR	NA	NA	NA	Normal	Hearing loss
24	16.3	>5	68	NA	DA+LAR	NA	NA	NA	Normal	Hearing loss
25	NA	3.9	206.7	256	LAR	1.4	NA	PR	NA	NA
26	37	12.1	62.1	NA	Surgery+LAR	4.6	NA	PR	NA	NA
27	11	8.1	35	1,105	LAR	NA	NA	NA	NA	NA
28	4.9	9.4	14.6	NA	RT+LAR	NA	NA	PR	Normal	NA
29	NA	NA	27–33	NA	Sandostatin+DA		NA	No	NA	NA
30	NA	NA	NA	NA	Surgery	NA	NA	NA	Bitemporal hemianopsia	NA
31	10.4	NA	46.1	NA	DA	–	No	NA	NA	
32	NA	NA	66	NA	Sandostatin+DA	NA	NA	NA	Normal	NA
33	5.4	NA	NA	NA	NA	NA	NA	NA	NA	NA
34	NA	NA	>200	NA	Surgery+DA	NA	NA	No	NA	NA
35	170	NA	190	2,500	Surgery	–	–	Dead	Visual defect	Hearing loss
36	5	NA	45.6	285	NA	NA	NA	NA	Right eye optic atrophy	Normal
37	98	NA	NA	NA	NA	NA	NA	NA	Normal	NA
38	NA	NA	NA	NA	Fractionated radiotherapy	NA	NA	NA	Left temporal hemianopsia	NA
39	5.3	NA	86.9	NA	DA	NA	NA	PR	Normal	Normal

GH, growth hormone; IGF-1, insulin-like growth factor-1; PRL, prolactin; ALP, alkaline phosphatase; Macro, macroadenoma; Micro, microadenoma; LAR, long-acting somatostatin analogue octreotide; DA, dopamine agonist; PEG, pegvisomant.

In the literature review, for patients who had been diagnosed with gigantism, for whom the final height was thoroughly documented, the final heights were 180 cm, 183 cm, and 169 cm, and the height Z-scores were 2.2, 2.6, and −0.9 for cases 9, 11, and 15, respectively. Additionally, the predicted heights were 172.5 and 175 cm for cases 11 and 15, respectively (the parental height was not mentioned in case 9). For patients who had been diagnosed with acromegaly, three of six patients (cases 1, 12, 14, 32, 38, and 39) had short stature (139 cm, Z-score= −3.2 in case 14; 147.5 cm, Z-score= −2.1 in case 32; and 140 cm, Z-score= −3.1 in case 39).

## Discussion

This study is the first case series to illustrate the clinical manifestations, treatment, and predicted or final height and the status of FD of combined GH excess and PP in the context of MAS patients. We found that the proportion of women with GH excess and PP of MAS was higher, whereas GH excess in MAS (14, 16) and “classic” acromegaly (59) affected almost equal proportions of women and men. This difference is likely due to PP affecting more female patients than male patients with MAS (8). In this case series, the number of MAS patients from PUMCH with gigantism was higher than the number with acromegaly, while approximately half of the literature review patients had gigantism. The average age at the acromegaly diagnosis in patients with MAS and PP in the literature review was 27.1 years, which is slightly lower than 30.1 years, the mean age of patients with acromegaly and MAS reported in a previous study (9); however, both of these ages were much younger than the median age of 40~50 years observed in “classic” acromegaly (60–62).

The data from PUMCH demonstrated that patients with a diagnosis of gigantism and PP have growth rate acceleration and skeletal maturation, and good control of both conditions leads to normalized GV and stabilized bone age. However, the data from PUMCH and a literature review showed that these patients had unaffected final heights. For those who were diagnosed with gigantism, the PAH Z-scores or final height Z-scores were higher than the predicted adult height Z-score. For patients diagnosed with acromegaly, one patient from PUMCH and approximately half of the patients reviewed in the literature had a short stature. Previous findings (15, 16) indicated that patients with a history of gigantism and PP achieve normal adult height despite early epiphyseal fusion, while those diagnosed with PP and acromegaly can end up with a significantly shorter adult height (63). The growth outcome in our cohort was that the height of MAS patients with PP and gigantism was higher than their predicted heights. This is probably because, in our cohort, PP was well controlled, while gigantism was partially relieved in some patients. This phenomenon indicates that sex hormones and GH have cumulative effects on the growth plate, and the time point at which GH excess appears is related to the final adult height.

The diagnosis and treatment of the coexistence of GH excess and PP is challenging. Under these circumstances, BA can better indicate the degree of sexual maturity. When height and BA are not parallel, we should consider comorbid gigantism. Laboratory screening for GH excess helps confirm the diagnosis. Good control of GH excess and PP is essential to reduce the growth rate and stabilize bone maturity.

Although patients with gigantism and PP in both our cohort and the literature review have unaffected final heights, early detection and treatment are still important because uncontrolled gigantism worsens CFFD. Bone turnover is increased in patients with GH excess, and they have increased levels of markers of bone formation and resorption (64). These biomarkers, particularly ALP, are usually related to the scope and intensity of bone involvement in MAS (65). Previous studies have already shown that uncontrolled gigantism is associated with the aggravation of CFFD and an increased risk of optic neuropathy (16–18) and hearing loss (19). Studies have also shown that early treatment during the pediatric period decreases the risk of these morbidities (15, 18) and results in lower serum ALP levels (14). In the present study, CFFD lesions were present in all patients from PUMCH, and polyostotic FD lesions were present in most patients, suggesting that they may be correlated with excess GH. Additionally, after effective treatment of GH excess, the visual field, hearing, and olfactory functions remained stable, and the ALP Z-score decreased after treatment. These results indicate that early successful treatment of GH excess might help ameliorate FD.

In addition to aggravating FD, GH excess is also related to impaired glucose tolerance, hypertension, cardiomyopathy, and an increased risk of tumors (22). Therefore, choosing the proper treatment is critical. Conventional treatments for GH excess include surgery, medications (somatostatin receptor ligands, Das, and the GH receptor antagonist pegvisomant), and radiotherapy. According to previous studies, medical therapy is considered the first-line treatment because pituitary surgery is technically difficult in patients with MAS because of the massive thickening of the skull base with FD (16, 35). However, technological progress has been made in this field in recent years, and we suggest that transphenoidal tumor resection under the guidance of neuronavigation might be feasible. An earlier cohort study from PUMCH revealed that transsphenoidal complete tumor excision with neuronavigational guidance is effective in patients with MAS with GH excess. In the present study, five of seven patients from PUMCH had undergone navigation-assisted transsphenoidal pituitary surgery, and two out of the five patients achieved full recovery. The remaining three patients had a partial response and then continued to receive long-acting somatostatin and bromocriptine. None of our patients had hypopituitarism after surgery. Therefore, from the experience of our center, navigation-assisted transsphenoidal pituitary surgery could be an effective and relatively safe choice for patients with MAS diagnosed with GH excess and PP. Radiotherapy is generally not advised because of the risk of fatal malignant transformation of CFFD in bones (66–68).

Uncontrolled PP can also cause psychological problems in children, and long-term exposure to high levels of estrogen elevates the risk of breast cancer and endometrial cancer. It has already been demonstrated that letrozole, a 3^rd^-generation aromatase inhibitor, and tamoxifen, an estrogen receptor modulator, are the most evidenced treatments (69). Medroxyprogesterone is also effective for controlling vaginal bleeding and breast development in the short term (13, 70). When secondary central PP develops, GnRHa is beneficial for blocking the hypothalamus-pituitary-gonadal (HPG) axis (71). In this study, the treatments for PP ranged from observation only to combinations of two medications. Letrozole and tamoxifen were the most frequently used. One patient developed secondary central PP and was treated with GnRHa for 3 months. Medroxyprogesterone was also used in combination with letrozole in one 6-year-old boy. During follow-up, we need to focus on changes in BA or the BA/CA ratio, not only changes in the growth rate, because the decline in the growth rate may only benefit from controlling excessive GH.

The co-occurrence of GH excess and PP in MAS is extremely rare. This study is the first to analyze the clinical features of this condition, and the diagnosis and treatment of both conditions pose considerable challenges. First, the diagnosis of gigantism may be overlooked because PP can also cause accelerated linear growth and atypical facial dysmorphia due to CFFD. Additionally, when evaluating the effectiveness of PP treatment in these patients, attention should be given to not only changes in GV but also changes in BA. A comprehensive consideration of GV, BA, and IGF-1 levels may lead to an accurate diagnosis and smooth follow-up.

However, this study still had several limitations. First, the sample size from PUMCH was small because the prevalence of this condition was extremely low, and patient 2 was lost of follow-up because of financial problem. We added 39 cases from the literature as supplementation. Additionally, when analyzing the association of GH excess with FD, we did not include a control group, and the severity of bone lesions and incidence of optic, hearing, and olfactory complications could not be compared with those in patients without GH excess.

## Conclusion

MAS with GH excess and PP is rare, and these patients present with increased growth velocity and advanced bone age. Early diagnosis and proper treatments are essential. The predicated or final height of patients with gigantism is not impaired, while the final height of patients with acromegaly is shorter.

## Data Availability Statement

The original contributions presented in the study are included in the article/supplementary material. Further inquiries can be directed to the corresponding author.

## Ethics Statement

This study is in compliance with the Helsinki Declaration, and the parents signed the informed consent form, with the approval of the Ethics Committee of Peking Union Medical College Hospital (Reference number: JS-1663). Written informed consent to participate in this study was provided by the participants’ legal guardian/next of kin.

## Author Contributions

XZ, LD, and HZ participated in the design of the study. XZ carried out the study and collected important background information. LW and HYa carried out literature research. LL, SC, and HP provided assistance for statistical analysis. YY, BX, KD, and RW provided assistance for surgical clinical data collection. FF and HYo helped with radiologic data collection. ZL participated in pathology data collection. All authors contributed to the article and approved the submitted version.

## Funding

This work was supported by a grant from the National Key Research and Development Program of China (No. 2016YFC0901501) and CAMS Innovation Fund for Medical Science (CAMS-2016-I2M-1-002).

## Conflict of Interest

The authors declare that the research was conducted in the absence of any commercial or financial relationships that could be construed as a potential conflict of interest.

## Publisher’s Note

All claims expressed in this article are solely those of the authors and do not necessarily represent those of their affiliated organizations, or those of the publisher, the editors and the reviewers. Any product that may be evaluated in this article, or claim that may be made by its manufacturer, is not guaranteed or endorsed by the publisher.
